# Loco-regional treatment with temozolomide-loaded thermogels prevents glioblastoma recurrences in orthotopic human xenograft models

**DOI:** 10.1038/s41598-023-31811-5

**Published:** 2023-03-21

**Authors:** Lisa Gherardini, Veronica Vetri Buratti, Mirko Maturi, Giovanni Inzalaco, Erica Locatelli, Letizia Sambri, Sara Gargiulo, Virginia Barone, Denise Bonente, Eugenio Bertelli, Silvia Tortorella, Lorenzo Franci, Antonio Fioravanti, Mauro Comes Franchini, Mario Chiariello

**Affiliations:** 1 Istituto di Fisiologia Clinica (IFC), Consiglio Nazionale delle Ricerche (CNR), Via Fiorentina, 53100 Siena, Italy; 2 Department of Industrial Chemistry “Toso Montanari”, University of Bologna, Viale Risorgimento 4, 40126 Bologna, Italy; 3 Core Research Laboratory (CRL), Istituto per lo Studio, la Prevenzione e la Rete Oncologica (ISPRO), Via Fiorentina 1, 53100 Siena, Italy; 4 University of Siena, Siena, Via Banchi di Sotto 55, 53100 Siena, Italy; 5 Department of Molecular and Developmental Medicine, University of Siena, Via Aldo Moro 2, 53100 Siena, Italy; 6 Department of Life Sciences, University of Siena, 53100 Siena, Italy; 7 Ospedale Maggiore (ASST), Largo Ugo Dossena 2, 26013 Crema, CR Italy

**Keywords:** Biotechnology, Drug discovery, Neuroscience, Cancer, Drug development

## Abstract

Glioblastoma multiforme (GBM) is the most aggressive primary tumor of the central nervous system and the diagnosis is often dismal. GBM pharmacological treatment is strongly limited by its intracranial location beyond the blood–brain barrier (BBB). While Temozolomide (TMZ) exhibits the best clinical performance, still less than 20% crosses the BBB, therefore requiring administration of very high doses with resulting unnecessary systemic side effects. Here, we aimed at designing new negative temperature-responsive gel formulations able to locally release TMZ beyond the BBB. The biocompatibility of a chitosan-β-glycerophosphate-based thermogel (THG)-containing mesoporous SiO_2_ nanoparticles (THG@SiO_2_) or polycaprolactone microparticles (THG@PCL) was ascertained in vitro and in vivo by cell counting and histological examination. Next, we loaded TMZ into such matrices (THG@SiO_2_-TMZ and THG@PCL-TMZ) and tested their therapeutic potential both in vitro and in vivo, in a glioblastoma resection and recurrence mouse model based on orthotopic growth of human cancer cells. The two newly designed anticancer formulations, consisting in TMZ-silica (SiO_2_@TMZ) dispersed in the thermogel matrix (THG@SiO_2_-TMZ) and TMZ, spray-dried on PLC and incorporated into the thermogel (THG@PCL-TMZ), induced cell death in vitro. When applied intracranially to a resected U87-MG-Red-FLuc human GBM model, THG@SiO_2_-TMZ and THG@PCL-TMZ caused a significant reduction in the growth of tumor recurrences, when compared to untreated controls. THG@SiO_2_-TMZ and THG@PCL-TMZ are therefore new promising gel-based local therapy candidates for the treatment of GBM.

## Introduction

Glioblastoma multiforme (GBM) is the most aggressive primary tumor of the central nervous system^1^. It has an incidence rate of around 2–3 over 100.000 persons in the population of developed countries, and a 5-year survival rate of approximately 5%^2^. The extremely difficult possibility of complete removal by surgery makes radiotherapy and chemotherapy key adjuvating approaches for tumor treatment^3^. Temozolomide (TMZ), a methyl-alkylating pro-drug causing mismatch during cell DNA repair mechanism, is the chemotherapeutic agent exhibiting the best clinical performance in GBM and, therefore, represents the Food and Drug administration (FDA)-approved first line chemotherapy intervention for these tumors^3^. Still, its efficacy is strongly limited by innate or acquired resistance of glioma cells to this drug^4^ as well as by an only limited ability to pass the blood–brain barrier (BBB)^5^. Consequently, TMZ needs to be administered frequently and at high doses^6^, often causing undesired side effects such as severe discomfort and myelosuppression^7^. Importantly, all these problems usually determine tumor recurrence within 6 months and there is no effective standard-of-care approach for a second-line therapeutic strategy^8^. Therefore, GBM treatment may really take advantage of innovative strategies of drug delivery, that could be able to ensure high concentration of active agents at the site of the tumor, to reach and kill frequent infiltrating cells and ensuring low systemic and organ toxicity. Localized delivery strategies, such as Convention Enhanced Delivery (CED)^9^ and focused ultrasonic irradiation^10^ are surely very promising approaches for GBM therapy^11^. On the other end, surgically implantable matrices releasing active agents may represent a valid alternative for GBM local treatment with the advantage of semi-controlled release of high amounts of chemotherapeutics. Gliadel wafers, loaded with carmustine, are the only FDA-approved agents for intracranial, peri-surgical GBM local treatment^3^. However, matrix rigidity reduces Gliadel effectiveness and may cause local damage to adjacent healthy tissues^12^. Consequently, the development of innovative biomaterials, able to more precisely fit and adapt to the surgical cavity, slowly release great amounts of the drug and, at the same time, protect it from degradation, may strongly benefit the therapeutic approach to this specific tumor^13^.

One of the latest frontiers proposed by the scientific community in order to optimize drug’s anti proliferative efficacy consists in the use of nanocarriers to simultaneously deliver anticancer drugs while protecting them from degradation and fast clearance from the body compartment^14^. Among such drug carriers, nanoparticles have been widely explored for therapeutic and diagnostic interventions^15^. However, the systemic administration of these carriers presents several limitations and drawbacks^15^ that can be overcome by a loco-regional administration route. In order to allow the localized administration of drug-loaded carriers, hydrogels have been selected as the most-promising candidates. Indeed, non-toxic and biodegradable polymers developed as “smart materials” are able to modulate chemical reactivity and matrices’ structural architecture and harden in response to chemo-physical stimuli such as pH, temperature or ion concentration^16^. Among them, chitosan-based hydrogels behave as temperature-sensitive hydrogels (or thermogels) as they are liquefied at room temperature but become gel at a physiological temperature (37 °C). Specifically, at low temperatures, hydrogen bonds are formed between hydrophilic groups of polymer chain and the system remains liquid. At this stage, polymers can be applied or delivered in liquid form to the body. Then, once in contact with the body districts, the polymer–polymer interactions get weaker while the interactions with secondary ligands strengthen as the temperature increases, normally, leading to sol–gel transitions at 37 °C^17^. Importantly, as this transition happens with simple temperature stimulation and without additional organic solvents or cross-linking agents, the risk of unexpected toxicity or side effects is strongly reduced, making such gels highly considered for pharmaceutical applications not only for their biocompatibility but also for their biodegradability, ability to carry hydrophilic and hydrophobic drugs, stability, and amenability^18^. Interestingly, chitosan/β-glycerophosphate (βGP) thermogels, after gelation, also rapidly loose excess βGP and water that do not contribute to the physical crosslinking of the gel, leading to a rapid loss of up to 35% total hydrated content^19^, which may support the observed shrinkage of these negative temperature-responsive hydrogels^18^, further contributing to their ability to adapt and precisely fit natural or surgically-created anatomical cavities. The chitosan hydrogels can be also grafted with other chemicals to modulate their performance and endurance^20^. For example, polyethylene glycol (PEG)-grafted chitosan is able to create a reversible phase transition gel with βGP and host biotechnologically engineered T lymphocytes for glioblastoma immunotherapy^21^. Overall, chitosan-βGP hydrogels are often used in the field of regenerative medicine as scaffolding to host regenerating biological elements such as stem cells but little evidences have been gathered about the potential of these matrices to treat cancer in vivo^22^ and none describing the preclinical investigation in orthotopic glioblastoma models, in particular in association with micro-encapsulation of TMZ.

Here, we investigated for the first time the potential of two different negative temperature-responsive chitosan-βGP-based thermogels (THG), containing TMZ-loaded mesoporous silica (SiO_2_) nanoparticles or polycaprolactone (PCL) microparticles, as innovative anti-proliferative local delivery formulations against GBM in vivo. The biocompatibility and efficacy of the two newly designed hydrogels were ascertained in vitro and in vivo, in an orthotopic murine model of human glioblastoma demonstrating, for both formulations, a significant reduction of tumor reoccurrence when compared to untreated controls undergoing only GBM surgical resection.

## Results

### Synthesis and characterization of TMZ-loaded microsystems

The exploitation of micro/nano carriers for the entrapment, protection and slow release of drugs is one of the latest frontiers in nanomedicine. For this reason, in the present work, two systems have been selected, synthesized and characterized. The most-promising carriers selected for the purpose of delivering TMZ into the brain compartment are PCL microparticles and mesoporous silica (SiO_2_) nanoparticles. Both these carriers have received long investigation in the scientific community and are well-known for their biocompatibility, high performance to entrap drugs and their well-defined release in a time-dependent manner^23,24^.

**Figure 1:**
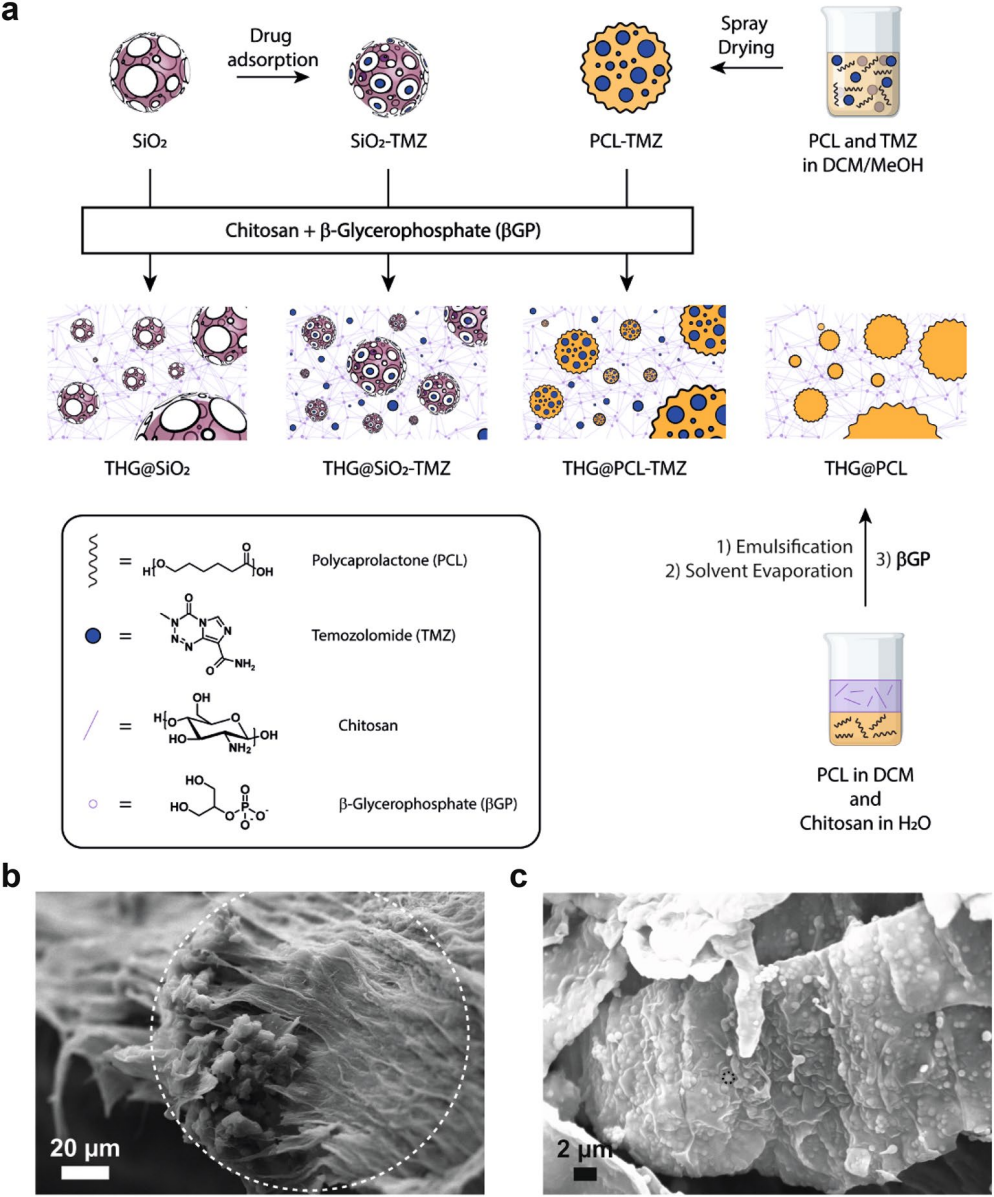
Explored strategies for loading TMZ in chitosan-based thermogels. (**a**) For SiO_2_-TMZ, the drug is adsorbed on mesoporous silica (SiO_2_) via electrostatic interactions, and the colloid is then dispersed in the thermogel matrix. For PCL-TMZ, the drug is dissolved together with PCL in a dichloromethane/methanol (DCM/MeOH) mixture and spray-dried to afford PCL-TMZ microparticles that physically entrapped the drug. The drug-loaded microparticles were then formulated in the chitosan/βGP thermogel (THG). (**b**) Scanning electron micrographs of freeze-dried THG@PCL-TMZ. (**c**) THG@PCL-SiO_2_-TMZ. TMZ-loaded microcarriers are highlighted with dashed circles.

We first encapsulated TMZ in SiO_2_ nanoparticles to obtain SiO_2_-TMZ (Fig. 1a). TMZ-loaded mesoporous silica (SiO_2_-TMZ) was synthetized adapting a procedure reported in literature, through which experimental conditions were optimized to achieve a suitable drug loading^25^. The drug-loading process allowed for the obtainment of 400 mg of SiO_2_-TMZ containing an initial amount of TMZ equal to 197 ± 3 mg of TMZ determined by High Performance Liquid Chromatography (HPLC), corresponding to a loading capacity of 49.3 ± 0.8 wt.% and a nearly quantitative encapsulation efficiency (≥ 97.0%), as reported in Table 1. Dynamic light scattering (DLS) revealed a mean hydrodynamic diameter of 636 ± 65 nm with a polydispersity index of 0.261 and a surface charge, measured as Zeta potential, equal to – 19.4 mV.

**Table 1:** Characterization of TMZ-loaded microcarriers.

	Size	ζ Potential (mV)	Encapsulation efficiency^a^ (%)	Loading capacity^a^ (%)
SiO_2_-TMZ	636 ± 65 nm^**a**^	− 19.0	≥ 97.0	49.3 ± 0.8
PCL-TMZ	107 µm	− 1.11	≥ 98.2	9.93 ± 0.11

^a^Data expressed as mean ± SD.

In parallel, PCL-TMZ was prepared by spray-drying of a combined solution of TMZ and PCL in dichloromethane/methanol (DCM/MeOH), and the experimental conditions were optimized to achieve a micronized powder with a satisfactory drug loading (Fig. 1a). The spray-drying process allowed for the obtainment of 3.02 g of TMZ-loaded microparticles (yield = 60.5%) containing 300 ± 3 mg of TMZ, as determined by HPLC, corresponding to a drug loading of 9.93 ± 0.11 wt.% and a nearly quantitative encapsulation efficiency (≥ 98.2%). Moreover, particle size analysis revealed a size distribution characterized by X_10_ = 23.8 µm, X_50_ = 84.8 µm, and X_90_ = 218 µm and a mean diameter by volume (VMD) equal to 107 µm. The surface charge measured as Zeta potential was equal to − 1.11 mV, which is expected for PCL polymer, since it did not present any charged functional group on its surface.

### TMZ-loaded chitosan/β-glycerophosphate thermogel formulation

Chitosan-based injectable gels with β-glycerophosphate (βGP) have attracted a lot of attention because of their ability to form thermosensitive gel delivery vehicles^26^. The effective interactions responsible for the gelation of chitosan/βGP mixture are: (1) βGP salts acts as a reducing electrostatic repulsion agent, increasing the hydrogen bonding among the chitosan chains; (2) electrostatic attractions between chitosan and βGP; (3) the enhancement of chitosan-chitosan hydrophobic interactions due to the structural role of βGP on water. Rheological properties and gelation temperature can be decreased and varied in the range of 32–37°C by increasing chitosan deacetylation degree, increasing βGP concentration, increasing pH (6.8–7.4), or reducing chitosan molecular weight.

We formulated TMZ-loaded chitosan-based thermogels using two different encapsulation approaches based on mesoporous silica (THG@SiO_2_-TMZ) or PCL (THG@PCL-TMZ) (Fig. 1a), to compare their performances in the delivery of TMZ for the therapy of GBM.

Scanning electron microscopy was performed on a freeze-dried portion of the prepared gels, revealing for both preparations the presence of a continuous network of chitosan/βGP in which the TMZ-loaded particles were embedded (Fig. 1b, c). Moreover, scanning electron microscopy images of THG@PCL-TMZ showed the presence of large spherical polymeric aggregates (with a diameter of 130 µm), while the images of THG@SiO_2_-TMZ displayed smaller particles (around 0.6 µm), in agreement with the particle sizes determined by DLS (for SiO_2_) and particle size analysis (for PCL).

Drug concentration in both gels was kept constant and equal to 5.38 mg/mL at the time of preparation. A preliminary analysis aimed at determining the extent of TMZ degradation in water in the 7 days intercurring between the drug encapsulation step and the moment in which the thermogel was injected into the mouse brain, revealed a TMZ loss of 35% over 7 days. With this information, it was possible to estimate a final active drug concentration, at the time of use, of 3.5 mg/mL.

Drug release tests were performed by dipping a dialysis membrane containing the thermogel into sterile PBS solution at 37 °C and measuring the released TMZ content over time. However, this approach did not allow for the obtainment of satisfactory release profiles, since TMZ degradation in PBS at 37 °C has demonstrated to be faster than the drug release itself.

In order to evaluate the mechanical behavior of the prepared thermogels upon needle injection, their viscosity was evaluated at 25 °C as a function of applied rotational shear, to evaluate their rheology at room temperature, when they are still in the liquid form and they need to be able to flow into a needle for administration (Fig. S1). Both thermogels displayed a clear shear-thinning behavior, since their viscosity was observed to decrease significantly upon increasing shear rate. This property suggests that the hydrogel can be injected through a needle, since the higher shear caused by the low diameter of the needle will cause a decrease in the hydrogel viscosity, allowing it to flow through. In addition, PCL-loaded thermogels are characterized by slightly higher viscosities throughout all the explored shear rate range, which is expected due to the presence of a higher amount of inert polymeric material compared to thermogels loaded with silica.

### Efficacy of TMZ and toxicity profile of the carrier hydrogels on the glioblastoma cell model, in vitro

**Figure 2:**
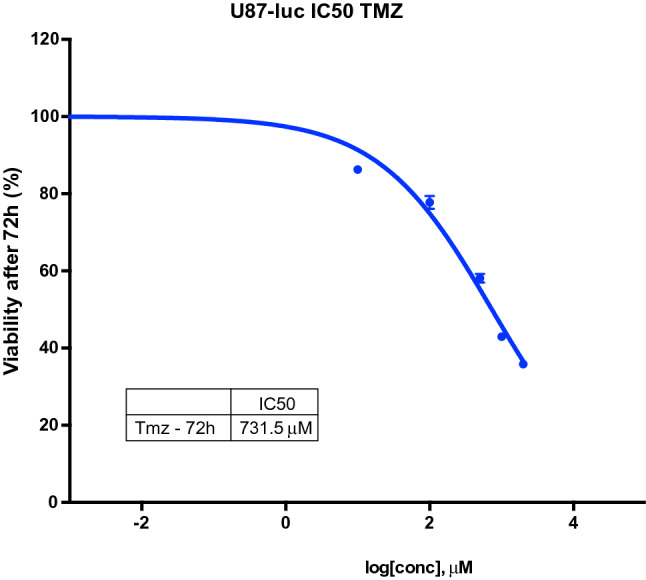
IC_50_ for TMZ on cell viability in U87 MG-Red-FLuc human glioblastoma cells by cell counts. The concentration-dependent inhibitory dose-curve data was plotted as log percentage inhibition normalized to controls with applied curve non-fit calculated using GraphPad Prism. Results are shown as the mean of at least three independent experiments ± SEM.

**Figure 3:**
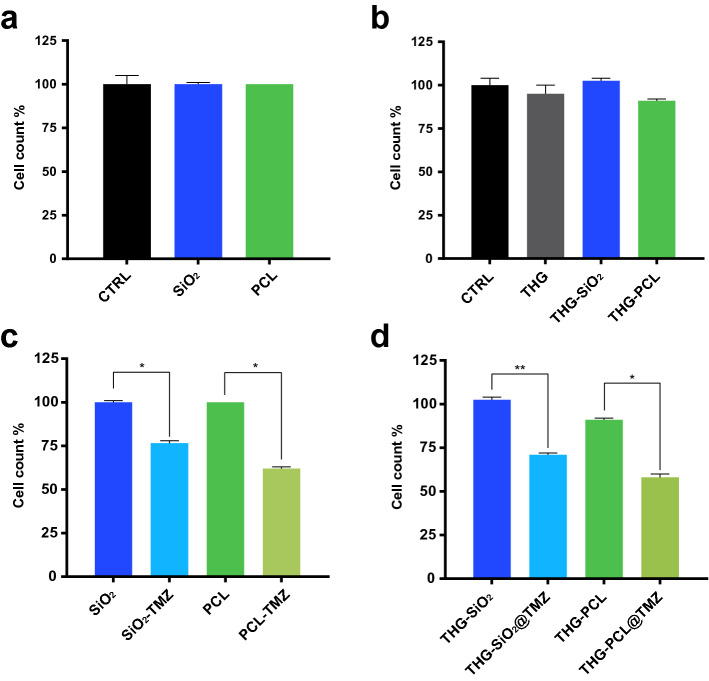
In vitro effect of the isolated components and of assembled new gel formulations on Glioblastoma cells. (**a**) U87-MG-Red-FLuc cells were treated with cell medium (CTRL), mesoporous SiO_2_ nanoparticles (SiO_2_) and polycaprolactone microparticles (PCL) for 24 h, causing no significant reduction in viable cells number. (**b**) U87 MG-Red-FLuc cells were treated with cell medium (CTRL), the empty thermogel (THG), and the thermogels containing mesoporous SiO_2_ nanoparticles (THG@SiO_2_) and polycaprolactone microparticles (THG@PCL) for 24 h, causing no significant reduction in viable cells number. (**c**) TMZ-containing SiO_2_ nanoparticles and PCL microparticles efficiently release active drug into the cell medium to induce cell death after 24 h treatment in U87 MG-Red-FLuc cells (*p < 0.05). (**d**) THG@SiO_2_-TMZ and THG@PCL-TMZ efficiently release active TMZ into the cell medium to induce cell death after 24 h treatment in U87-MG-Red-FLuc cells (*p < 0.05; **p < 0.005).

To track tumor growth in an orthotopic mouse model for glioblastoma, we decided to use commercially available human cancer cells expressing a very bright red-shifted luciferase (Red-FLuc), U87-MG-Red-FLuc cells (https://www.perkinelmer.com/product/ivisbrite-u87mg-red-f-luc-bw124577). These cells were first verified for sensibility to TMZ treatment, by application of step-increased concentrations of TMZ to the cell culture for 72 h, to evaluate of the IC_50_ value. The TMZ IC_50_ for these cells was 731.5 mM (Fig. 2), in the range of values already reported in analogous conditions^27^. Importantly, we also verified the absence of toxic effects exerted by the SiO_2_ and PCL components and by the THG, THG@SiO_2_ and THG@PCL hydrogels on the same cellular system, to ensure that effects on viability could be ascribed to the specific TMZ drug. Indeed, both SiO_2_ and PCL alone (Fig. 3a) or loaded into the thermogels (THG-SiO_2_ and THG-PCL), as well as the THG per se (Fig. 3b) did not exert any significant effect on glioblastoma cell viability. Conversely, treatment with SiO_2_-TMZ and PCL-TMZ (Fig. 3c) as well as the corresponding loaded thermogels (THG@SiO_2_-TMZ or THG@PCL-TMZ) (Fig. 3d) significantly affected cells viability, suggesting that these anti-cancer formulations are able to release of TMZ in its active chemotherapeutic form.

### Biocompatibility of the thermogel in vivo

**Figure 4:**
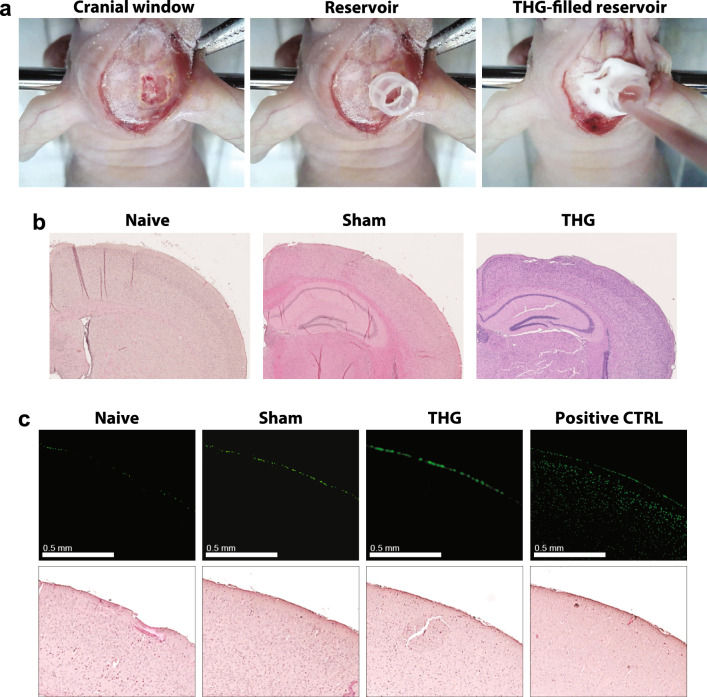
THG induced no toxic reaction after locally treatment of the brain tissue for 24 h. (**a**) A cranial window in the skull was created to expose the brain tissue for direct application of THG thermogel. To apply the gel, a customized bottomless plastic reservoir was fixed to the skull, filled with fluid gel (4 °C), closed and secured on the mouse head. (**b**) Representative images of histological investigation of brains from naïve, sham and THG-treated mice. H&E on coronal sections from each of experimental case revealed no major sign of adverse cellular and structural change. (**c**) TUNEL reactions on brains from naïve, sham and THG-treated mice (upper panel) and the same slides after H&E staining (lower panel). No apoptosis was detected in naïve, sham and THG-treated specimens. Positive control (CTRL) was obtained by treating the slices with DNase I. TUNEL unspecific fluorescence on the surface of brains, due to the presence of cerebral membranes, is present in all treated samples as well as in naïve sections.

In vitro data indicated that none of the gels used for the formulations was likely to induce cell toxicity. To adhere to the Reduction guidelines as formulated by “Animal Research: Reporting of In Vivo Experiments” (ARRIVE), we next tested empty THG for bio-toxicity also in vivo, as a reference for both THG-derived gel formulations (Fig. 4a). Qualitative histological investigation on brains treated for 24 h with THG showed no sign of anatomical changes with respect to sham (no gel application) animals subjected to craniotomy or naïve animals (Fig. 4b). Importantly, no sign of apoptosis was noticed on brains treated with THG, when scored by Terminal dUTP Nick End Labeling (TUNEL) assay (Fig. 4c), confirming brain tissue biocompatibility of the used thermogel.

### In vivo efficacy of gel-based TMZ local treatment using a GBM resection and recurrence mouse model

Together with radio- and chemotherapy, surgical resection is the standard of care approach for primary glioblastomas^3^. Still, the difficulty in obtaining complete surgical removal of the lesion and the strong invasiveness of cancer cells even in the early phases of tumor development make recurrences almost sure and, unfortunately, very rapid. As our approach is specifically aimed at preventing development of GBM recurrences, we therefore decided to use a specific resection and recurrence mouse model in which the orthotopic human U87-MG-Red-FLuc-derived tumor is surgically excised^28^ and, after application of the therapeutic gel, the size of the recurrence can be scored by optical bioluminescence imaging (BLI) thanks to Red-FLuc expression in GBM cells. A week after the injection of U87-MG-Red-FLuc cells into the mouse striatum, tumors underwent partial resection. Based on our previous set-up experiments and available published data^29^, the void in the tissue cavity was filled with 10 μL of cooled fluid gel which, upon hardening with increasing temperature up to 37 °C, was qualitatively confirmed to completely adhere to the cavity boundaries. Based on our previous estimation of a final active drug concentration of 3.5 mg/mL (see above), we calculated that the 10-μL amount of applied gel contained 35 μg of TMZ, corresponding to a final dose of approximately 1.75 mg/kg. Importantly, this dose is in line with previous reports where efficacious gel-contained TMZ doses ranged between 0.6 and 4.75 mg/kg, when used in equivalent mouse and rat GBM preclinical systems^30–32^.

**Figure 5:**
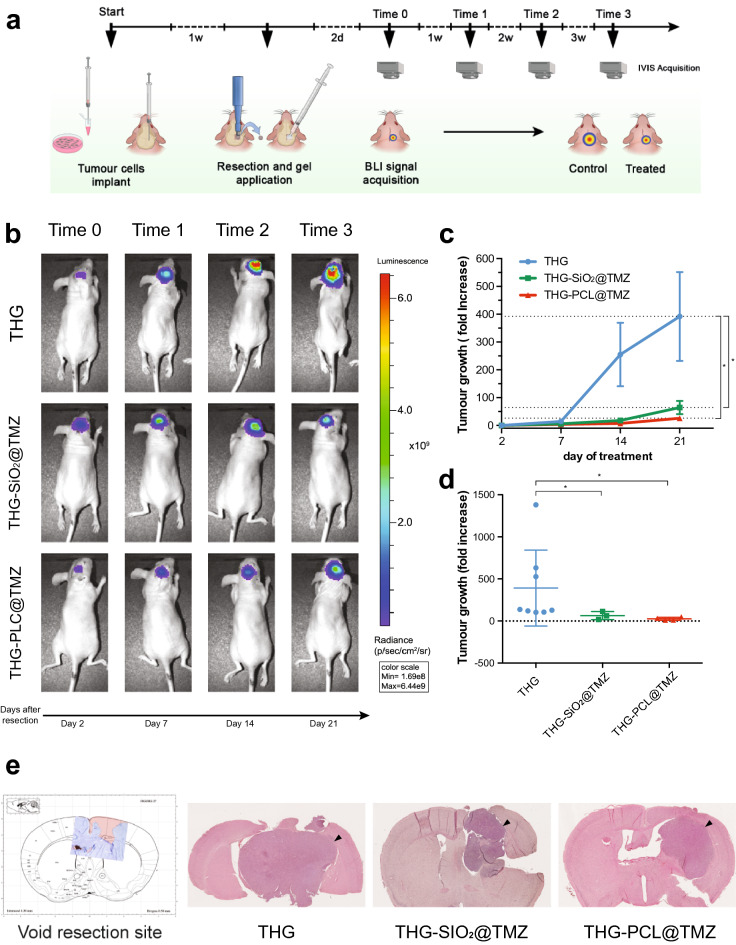
In vivo efficacy of TMZ-based therapeutic thermogels using a GBM resection and recurrence mouse model. (**a**) Experimental timeline for studying the in vivo anticancer activities of gel-based local glioblastoma treatments. Two independent experiments comparing empty THG with THG@SIO_2_-TMZ and THG@PCL-TMZ, respectively, were performed (n = 5). Results were next merged resulting in n = 8 for THG, n = 3 for THG@SIO_2_-TMZ and n = 4 for THG@PCL-TMZ, considering drop-outs due to the severity of the procedure. (**b**) Representative BLI images of mice bearing U87-MG-Red-FLuc implantation in the subcortical striatal region. After primary tumor resection, THG@SIO_2_-TMZ, THG@PCL-TMZ or empty THG were applied. (**c**) Tumor growth was directly related to the increment of luminescence signal normalized to baseline (day 2 after resection, corresponding to Time 0 in panels A and B) and recorded at days 7 (Time 1), 14 (Time 2) and 21 (Time 3) after resection, in THG, THG@SiO_2_-TMZ and THG@PCL-TMZ groups. Tumor growth was expressed as fold increase [Lum(tx)−Lum (t0)/Lum (t0)]. THG@SIO_2_-TMZ or THG@PCL-TMZ reduced tumor recurrences when compared to empty THG treatment (*p < 0.05; two-ways ANOVA p < 0.05). (**d**). Luminescence signals from control (empty THG) animals on day 21 are significantly higher (*p < 0.05; Kruskal–Wallis test p < 0.004). (**e**) Site of the resection in relation to the Paxinos representation of brain coronal section at the injection coordinate (− 0.5 mm). Representative images of H&E-stained brain sections collected 21 days after tumor resection and gel application from mice receiving either THG@SIO_2_-TMZ, THG@PCL-TMZ or empty THG (control animals) show the growth of the tumor mass to the areas adjacent to the cancer cells injection site.

With our system, the gel remained therefore in place and in contact to the whole surrounding tissue, homogenously releasing the TMZ content for the entire duration of the experiment. In each mouse, tumor size was directly correlated to the bioluminescence detected from Red FLuc-tagged cancer cells growing intracranially and monitored at baseline (calculated as the amount of residual tumor cells after surgery, measured 2 days after resection of the tumor) and then weekly for three weeks after gel application (Fig. 5a). At the end of the 3-weeks treatment, the bioluminescence signal from mice treated either with THG@SiO_2_-TMZ or THG@PCL-TMZ was significantly lower than in controls (Fig. 5b–d). Specifically, after 3-weeks treatment, luminescence signal (i.e. tumor growth) in the control group of mice receiving empty THG increased 392.00-fold, while mice treated with THG@SiO_2_-TMZ and THG@PCL-TMZ showed much smaller increases, i.e. 64.02-fold and 25.99-fold, respectively (Fig. 5c, d; Table 2), overall showing significantly delayed tumor recurrences.

**Table 2:** The effect of the different treatments on tumor growth.

Treatments	Luminescence signal (Lum)		
	Baseline	Final	Growth (fold increase)
THG	1.25 × 10^8^ ± 4.79 × 10^7^	2.07 × 10^10^ ± 5.28 × 10^9^	392.00
THG@SiO_2_-TMZ	3.04 × 10^8^ ± 1.26 × 10^8^	1.83 × 10^10^ ± 9.56 × 10^9^	64.02
THG@PCL-TMZ	5.42 × 10^8^ ± 1.73 × 10^8^	9.96 × 10^9^ ± 1.44 × 10^9^	25.99

The tumor growth is calculated as mean of bio-luminescence ± Standard Error of Mean (SEM) and displayed as fold increase [r Lum(tx)−Lum (t0)/Lum (t0) of light emission (Lum)].

The effect of THG@SiO_2_-TMZ and THG@PCL-TMZ treatments, compared to controls, was also confirmed by Hematoxylin/Eosin (H&E) stain investigation (Fig. 5e). In addition, the lack of systemic toxicity of TMZ treatment, delivered by means of both gels, was assessed by monitoring the body weight of animals undergoing different therapies: no significant body weight loss was observed in mice treated with any of the active formulations (Fig. S2), suggesting low systemic toxicity of TMZ when used as hydrogel-based local chemotherapy.

## Discussion

A wide range of anti-cancer drugs have been found to kill GBM cells in vitro, possibly even more efficiently than TMZ. Still, the vast majority of them are often doomed to fail in clinical trials because of their inability to cross the BBB. TMZ itself is far from being the ideal drug for systemic administration. Indeed, only 20% reaches the brain^5^, therefore requiring administration of high amounts, most of which only contribute to extremely deleterious systemic side-effects, ultimately causing frequent discontinuation of the necessary therapy regimen. To work around this problem, implantable delivery systems have gained special attention to control the release of drugs and localize treatments in specific sites next to a targeted tissue to create much higher local drug concentrations. Importantly, a postoperative implant for locoregional treatment of GBM, i.e., Gliadel Wafers, has been already developed and FDA-approved, but important side effects, mainly ascribed to the stiffness of the support and the use of carmustine as active molecule, have strongly limited its use^33^. Therefore, formulation of a “soft” support consisting of biocompatible gels is currently regarded as a possible solution for some Gliadel’s side effects. In this context, local delivery of TMZ via a biologically inert carrier composed of dextran phosphate (Temodex) has been already demonstrated to prolong survival in glioma patients when combined to standard therapy^34^, although very limited information is currently available regarding potential side effects of this approach (https://clinicaltrials.gov/ct2/show/NCT04967690).

The recent evolution of these types of “devices” is represented by the injection of gel forming solutions^35^ that can include a variety of therapeutic agents such as hydrophobic drugs, growth factors, and cells that can be directly delivered into the targeted site by a simple injection of a liquid formulation, which undergoes thermal gelation at body temperature^36^. Preclinical application of injectable thermo-sensitive gels in cancer therapy has been widely explored with encouraging results^37^. Among them, large importance was given to thermo-sensitive gels created by combining PLGA and PEG polymers in various forms and amounts, with particular emphasis on the tri-block PLGA-PEG-PLGA copolymer, with many studies showing interesting results in preclinical models; unfortunately, these polymers, being totally synthetic, still present doubts as well as unsolved biocompatibility and side effect issues, and no further studies are nowadays present in the literature. On the contrary, very few examples of totally bio-based and bio-derived thermo-responsive hydrogels have been developed and evaluated so far^13^, but none of them evaluated in vivo in orthotopic GBM models. Here, we implemented such a novel therapeutic formulation to enhance drug bioavailability at cancer lesions, based on a versatile chitosan-βGP thermogel platform which, being liquid before contact with the body, offers easier handling and positioning into the lesions. In particular, among thermo-sensitive gel systems, chitosan-βGP has been employed mostly in regenerative medicine^38,39^. Several research groups carried out the synthesis and characterization of chitosan-based nanocomposite injectable hydrogels^40^ but very few reported the pharmacokinetic profile of anticancer drugs incorporated into nanoparticle contained in hydrogels^41^. Chitosan hydrogel was used for treating glioblastoma in xenograft model, where the gel containing chemotherapy was injected on flank tumor bed^42^. In this case, however, the gel lacked the peculiarity of being thermo-sensitive, fundamental feature to be used as intracranial delivery. Chitosan/βGP thermo-sensitive gel was used to deliver ellagic acid (EA) to C6 glioma cells, in vitro^43^. However, in this case, the full potential of thermogels as tissue-adaptive material was not fully explored in vivo.

Importantly, we investigated the difference in the effectiveness of encapsulated TMZ loaded-mesoporous nanoparticles (MSNPs) and TMZ-spray-dried polycaprolactone (PCL) microparticles. In general, MSNPs present well-defined and tunable physicochemical properties, including particle and pore size, pore volume, surface area, volume area, pore structure, and surface functionality^44^. Characterized by a large chemical surface area and a controlled and adjustable porous structure, MSNPs can provide cavities that can host and release a great variety of biomolecules and therapeutic agents and acts as an excellent nano-platform for drug delivery systems^45^. Moreover, evidences of brain biocompatibility and safety of mesoporous silica-based materials for local implantation are already available^46,47^.

On the other hand, spray-dried polycaprolactone (PCL) microparticles have found extensive application for drug encapsulation and sustained release thanks to their biocompatibility and biodegradability^48^. Our study also demonstrated that rheological adaptability of thermosensitive gels containing SiO_2_-TMZ and PCL-TMZ allowed their use in vivo, providing the largest contact with resected wall interface at tumor site, optimizing TMZ local release and efficacy. Moreover, our results demonstrate that either THG@SiO_2_-TMZ or THG@PCL-TMZ can be effective as single anticancer treatment. We found that the tumor size recurrence was reduced by 6- to 13-fold when treated with THG@SiO_2_-TMZ and THG@PCL-TMZ thermogels, respectively. We hypothesize that the dramatic effect of highly concentrated local TMZ delivery might lead to reduced ability of the cells to develop resistance to TMZ, as also shown in human patients treated by local delivery of TMZ via a biologically inert carrier (Temodex)^34^.

Strengths and limitations of used cell lines and animal models also deserves additional consideration. Regarding the cellular and animal model system used in this study, we believe that the U87-MG glioblastoma resection model in athymic nude mice, originally described by Danhier and coll.^28^, currently represents the best approach to evaluate novel pharmacological approaches in the context of GBM surgical resection, which is the mainstay in the standard care of patients with GBM today. Importantly, as in most cases incomplete resection is the cause of recurrence in affected individuals^49^, we believe that this approach is particularly fit for modeling the effects of local drug delivery in preventing tumor recurrences, besides creating the space to locate the gels within the postoperative brain cavity. Clearly, the necessity of using immunocompromised mice to avoid rejection of human cancer cells prevented us from reaching any conclusion about the participation of the immune system to the efficacy of the system, but this was not an aim of our study. Also, once identified the most promising therapeutic thermogels, it will be important to test them on a panel of primary GBM cell lines, fingerprinted for their specific molecular characteristics, to consider the heterogeneity of human glioblastoma and evaluate potential tumor-specific responses. Importantly, availability of GBM cells engineered to express high levels of a luciferase gene allowed us to score in vivo the growth of the tumor with evident advantages at the experimental and ethical levels. It is also important to point out that, for logistic reasons, we have standardized a 1-week timeframe between gel preparation and final utilization in the mice, which could determine a reduction of the effective TMZ dose available in the gel at the moment of its application in vivo. Indeed, TMZ is subjected to hydrolysis in aqueous environments because of its reactivity towards water molecules^50^. Nonetheless, we have “modeled” the storage conditions of the drug in the hydrogel environment to be able to precisely predict its drug concentration at the time of injection, i.e. 7 days after drug formulation.

Although several studies have shown a delay in the onset of recurrences following local treatment with temozolomide (or few other drugs)^51^, the big challenge in this field is necessarily to avoid the onset of recurrences at all. While we used TMZ as a model drug for GBM pharmacological therapy, we believe that a real cure, able to completely avoid onset of recurrences, will necessarily pass through the use of drugs more efficacious than TMZ, possibly targeted drugs inhibiting specific GBM drivers/oncogenes. Our approach, therefore, aims at developing a new class of interchangeable and biocompatible thermogels able to re-evaluate more innovative classes of GBM drugs in vivo, independently of their ability of passing the BBB. In the long term, we hope that local delivery of chemotherapeutic drugs into the brain will possibly overtake the current approach of testing systemically-administered promising anti-GBM drugs in clinical trials without considering that most of them will not pass the BBB in sufficient amounts to result therapeutically efficacious.

In conclusion, we proved that the development of smart materials matrices constituted by chitosan-β-glycerophosphate-based thermogels (THG) hosting drug-enriched mesoporous silica nanoparticles or polycaprolactone microparticles may represent a valid approach for local targeted therapy of GBM. We provided evidence of potential translational development of our two innovative formulations for the local treatment of intracranial diseases beyond the BBB and we speculate that THG@SiO_2_ and THG@PCL drug delivery platforms are tunable for different clinical settings.

## Methods

### Chemicals

Medical grade chitosan (MW 33 kDa, degree of deacetylation 91.5%, viscosity 75 mPa s 20 °C) was purchased from Chitocean (Newfoundland, Canada). Temozolomide was purchased from Tokyo Chemical Industry (Tokyo, Japan) and used as received**.** Mesoporous silica nanoparticles colloidal aqueous dispersion (15 wt.%, pore size 4 nm, 0.5 µm particle size) was purchased from Sigma-Aldrich (St. Louis, MO). All the other chemicals are purchased from Sigma-Aldrich (St. Louis, MO) and used as received. All aqueous solutions were prepared with deionized (DI) water obtained using an ultrafiltration system (Milli-Q, Millipore) with a measured resistivity above 18 MΩ/cm.

### Determination of TMZ content by HPLC

Standard solutions of TMZ have been prepared in the 10–180 µg/mL concentration range by appositely diluting in water a stock TMZ solution containing 147.1 mg of TMZ in 25 mL of a 1:1 mixture of MeOH and diluted aqueous HCl (pH = 4.5). After microfiltration of the prepared solutions, they have been injected in HPLC by adapting a method described previously^52^. In particular, HPLC analysis was performed on an Agilent LC1120 system equipped with a manual injector using a C-18 column (300 mm × 4.6 mm i.d., 5 μm particle size) and a methanol/acetic acid (0.03% in water) 35/65 as mobile phase. The freshly prepared mobile phase was filtered through a 0.20 μm pore size nylon membrane filter and pumped in an isocratic mode with a flow rate of 1 mL/min. The elution of the analyte was monitored at a wavelength of 330 nm. The integrals of the peak corresponding to TMZ elution (retention time = 3.6 min) were plotted against the corresponding concentration and linearly fitted (Fig. S3). This calibration curve was employed for the determination of TMZ content in all the drug-loaded systems.

### TMZ adsorption on mesoporous silica (SiO_2_-TMZ)

SiO_2_-TMZ was synthetized adapting a procedure that is already reported in literature^53^. In a 150 mL round-bottomed flask 200 mg of temozolomide (TMZ, 1.030 mmol) were dissolved in 10 mL of dimethyl sulfoxide. After the addition of 95 mL of water, 1.33 mL of SiO_2_ mesoporous nanoparticles (MSNPs) aqueous solution (15 wt.%) was added dropwise. The reaction was performed at room temperature overnight under moderate stirring conditions. After purification, performed by consecutive steps of centrifugation (3000 rpm for 15 min) with Amicon® Ultra Centrifugal Filters (MWCO 100 kDa). The encapsulation efficiency was calculated as the ratio between the mass of TMZ detected in the purified nanosystem by HPLC as described in the previous paragraph and the total amount of drug employed in the encapsulation step. On the other hand, the loading capacity was calculated as the ratio between loaded TMZ and the total carrier mass.

In addition, SiO_2_-TMZ were dispersed in water and stored at + 4 °C. The encapsulation efficiency was evaluated by subtractive method, following the determination of the TMZ concentration in the waste waters after centrifugation.

$${\text{Encapsulated TMZ}} = {\text{Total amount of TMZ }}\left( {200{\text{ mg}}} \right) - {\text{TMZ in waste water }}\left( {\text{determined by HPLC}} \right){ }$$

### TMZ encapsulation in PCL microparticles by spray-drying (PCL-TMZ)

Temozolomide (500 mg) and PCL (Mw = 14 kDa, 4.5 g) were added to 250 mL of 2:1 CH_3_OH/CH_2_Cl_2_ and stirred until clear. This solution was sprayed on a Buchi B-290 spray dryer fitted with a two-fluid nozzle and a standard Buchi cyclone (outlet temperature = 30 °C, atomization pressure = 1 bar, flow rate = 2 mL/min) by Upperton Pharma Solutions (Nottingham, UK). In order to evaluate the TMZ content of the spray-dried product, 20 mg of micro-powder with 2 mL of aqueous HCl (pH = 4.5) by vigorously vortexing for around 20 min. After centrifugation (5 min, 10 krpm) the supernatant was separated from the solid polymer, microfiltered and injected in HPLC to determine encapsulation efficiency and drug loading. As before, the encapsulation efficiency was calculated as the ratio between the mass of TMZ detected in the purified nanosystem by HPLC as described in the previous paragraph and the total amount of drug employed in the encapsulation step. On the other hand, the loading capacity was calculated as the ratio between loaded TMZ and the total microcarrier mass.

Particle size analysis of the micronized powder was performed using a Sympatec HELOS particle size analyzer with a cuvette wet disperser, and an R5 lens (range 0.5–875.0 μm). A powder sample was taken, and a paste was produced using DI water and (1%) polysorbate 20. The cuvette was filled with DI water and 1 wt.% polysorbate 20 solution. The sample paste was introduced into the cuvette up to an obscuration of 20%. Dispersal was achieved using stirring at 800 rpm. From the particle size distribution, X_10_, X_50_ and X_90_ size values were extracted as representing respectively the upper size limit of 10, 50 and 90% of the measured particles sizes. Moreover, the volume mean diameter (VMD) was determined from the size distribution by the total volume of the sample divided by the number of particles, thus representing the diameter of a particle whose volume, if multiplied by the total number of particles, will equate all the sample’s volume.

### Preparation of Chitosan acetate

Water-soluble chitosan acetate polyelectrolyte was prepared by dissolving 1 g of chitosan in 100 mL of 1 vol.% acetic acid aqueous solution, followed by purification by dialysis against DI water [Molecular weight cutoff (MWCO) 3 KDa] to remove excess of acetic acid until pH = 7 was reached. The obtained chitosan acetate solution was then freeze-dried and employed for the preparation of the injectable thermogels.

### Preparation of SiO_2_-TMZ-loaded Chitosan/β-glycerophosphate (βGP) thermogels (THG@SiO_2_-TMZ)

For the preparation of 10 mL of injectable thermogel, 160 mg of chitosan acetate were dissolved in 6.9 mL of sterile DI water and 110 mg of SiO_2_-TMZ in 1.1 mL of water were then added to the obtained solution After thorough mixing and homogenization, the mixture was cooled to 0 °C using an ice bath, followed by the addition of a solution composed of 190 mg of sodium βGP dissolved in 2 mL of water. The liquid formulation was then loaded in single-use syringes and stored at + 4 °C until use.

### Preparation of PCL-TMZ-loaded Chitosan/βGP thermogels (THG@PCL-TMZ)

The chitosan-based thermogels were fabricated in sterile conditions and all the starting materials were autoclaved before use. For the preparation of 10 mL of injectable thermogel, 160 mg of chitosan acetate were dissolved in 6.9 mL of sterile DI water and 538 mg of PCL-TMZ in 1.1 mL of water were then added to the obtained solution. After thorough mixing and homogenization, the mixture was cooled to 0 °C using an ice bath, followed by the addition of a solution composed of 190 mg of sodium βGP dissolved in 2 mL of water. The liquid formulation was then loaded in single-use syringes and stored at + 4 °C until use.

### Preparation of SiO_2_-TMZ-loaded Chitosan/βGP thermogels

For THG@SiO_2_, unmodified mesoporous silica (55 mg in 1.1 mL of water) was added to the chitosan acetate solution (160 mg in 6.9 mL of water) and the thermogel was then prepared by adding sodium βGP, as described for the other samples.

### Preparation of PCL-loaded Chitosan/βGP thermogels

THG@PCL was prepared by emulsifying a solution of PCL in dichloromethane (484 mg in 1 mL) with a chitosan acetate solution (160 mg in 8 mL of water) by tip-probe sonication (40% amplitude, 3 min) followed by the evaporation of the organic solvent by heating at 50 °C for 1 h. The thermogel was then prepared by adding sodium βGP, as described for the other samples.

### Characterization of Chitosan/βGP thermogels

Scanning Electron Microscopy on freeze-dried thermogels was performed using a Zeiss EVO LS LaB6 scanning electron microscope operating at 5 k. Rheological experiments were performed on an Anton-Parr MCR102 modular compact rheometer with a DPP25-SN0 geometry, indicating a double plate geometry with a diameter of 25 mm. Rotational viscosity experiments (controlled shear rate tests, CSR) were performed on liquid thermogel samples at 25 °C by varying the shear rate from 0.1 to 1000/s.

### Cell culture

Glioblastoma U87-MG-Red-FLuc cell line for In Vivo Imaging System (IVIS) were purchased by Perkin Elmer. U87-MG-luc Red were cultured in Eagle’s Minimum Essential Medium (EMEM; ATCC, USA), supplemented with 10% fetal bovine serum (Gibco, USA), 2 mM l-glutamine, 100 units per mL penicillin–streptomycin (EuroClone) and maintained at 37 °C in an atmosphere of 5% CO_2_/air.

### Cell viability assay

To test TMZ effect on U87-MG-Red-FLuc, cells were seeded at 3 × 10^4^ cells/well density in 12‐well plates, in triplicate. After 24 h, cells were treated with different concentrations of TMZ in a range from 0 to 2 mM (0.01, 0.1, 0.5, 1.0, 2.0 mM). After 72 h incubation, cells were washed in PBS and harvested. Cell number of each sample was determined with Z2 Coulter Counter (Beckman Coulter), in triplicate. Data about cell viability were plotted in GraphPad Prism 8.0 software (GraphPad Software) to draw dose–response curve and to calculate the Inhibitory dose 50% (IC50).

### The effect of gel formulations in vitro

We tested the effect of SiO_2_, PCL, SiO_2_-TMZ, PCL-TMZ, THG, THG@SiO_2_ and THG@PCL, THG@SiO_2_-TMZ and THG@PCL-TMZ in U87-MG-Red-FLuc. Cells were seeded at 5 × 10^4^ cells/well density in 12‐well plates, in triplicate. After 24 h, cells were treated either with 1 mL of fresh medium alone (control) or 1 mL volume plus an amount of each of the treatments corresponding to in vivo conditions. For in vitro experiments TMZ concentration loaded in the gels was = 19.29 mM (3.744 mg/mL). After 24 h of treatments, the medium was removed, cells were washed Phosphate Buffer Saline (PBS), harvested and analyzed by microscopic visualization in a 100 µm deep Bürker counting chamber (Brand). Each experiment was set up in triplicate.

### Animal studies

#### Ethical statement

Experiments were conducted on opportunistic pathogen-free NMRI nude mice (Charles Rivers), female, six to seven weeks old, in accordance with EU Directive 2010/63/EU and Italian Ministry of Heath rules (approved projects: 09092018 RISS_161118). Experimental procedures were approved by the ethical committee of “Toscana Life Sciences (TLS) Animal Welfare Body”. All experiments were performed in accordance with relevant guidelines and regulations and follow the recommendations in the ARRIVE guidelines. Mice were maintained on standard laboratory food and water ad libitum, with a 12 h artificial light/dark cycle. In this manuscript, we did not measure death of the animals as an outcome measure, but we assessed the need to sacrifice the animal according to our declared score for humane endpoints.

### In vivo biocompatibility

The new gel-based formulation was applied onto the brain surface of healthy mice after craniotomy, to verify biocompatibility. A surgical incision of the scalp was performed on the midline and dissected from the periosteum, and a 3 mm^2^ dowel of skull bone was removed to expose brain surface. A plastic customized void reservoir was applied with dental cement onto the bone. Ten milliliters of gel were placed into the reservoir, to adhere to the tissue surface. The reservoir was capped and the wound was sutured with surgical glue. The same procedure was adopted in the sham-operated group. Animals were allowed to recover, and monitored up to 24 h. Paired aged naive animals were used as controls. For detecting biocompatibility, we used n = 3 animals. After euthanasia, brains were collected and imbedded 10% buffered formalin for 20 days and preserved in paraffin for histological analysis.

### Histological analysis

Immediately after removal, the brain was fixed in 10% buffered formalin for 20 days. The samples were then treated as previously described^54^. Briefly, after dehydration and paraffin embedding, serial 8 μm sections were cut within 1 mm of the tumor resection site with a microtome (Leica Microsystems), stained with Mayer's M&E and observed under a Nikon Eclipse E600 microscope.

A Terminal dUTP Nick End Labeling (TUNEL) assay was used to identify apoptotic cells according to the manufacturer’s instructions (In Situ Cell Death Detection Kit, Roche). Briefly, paraffin-embedded tissue sections were rehydrated and incubated in 0.1 M citrate buffer pH = 6 at 98 °C for 10 min. Then, after blocking in 20% normal bovine serum, TUNEL reaction was performed. Positive control was obtained by previously incubating slides with DNase I (1500 U/mL) for 10 min. Images were acquired by a NanoZoomer S60 Digital slide scanner, 20× objective (Hamamatsu).

### Reoccurring glioblastoma model by partial tumor resection

A 5 mm incision was made along the midline and the scull was exposed. A burr hole was performed into the skull at the right frontal lobe, 0.5 mm posterior and 1.75 mm lateral to the bregma using a manual drill (a 18G blunted needle). U87 MG-Red-FLuc glioblastoma cells (5 µL, 1 × 10^5^) were injected into the right striatum at a depth of 3.0 mm and at the rate of 1 mL/min using a 5 mL Hamilton syringe. Surgical wound was closed by surgical glue and animals were allowed to awaken and maintained under infrared heating lamp^28^.

On day 9 post-inoculation (day 2 post resection), mice were assigned to control (resection + empty thermogel) or treatment (resection + TMZ containing thermogel) groups by minimization according to tumor volume as measured by bioluminescence imaging. A biopsy punch surgical technique was next adapted from Bianco et al.^28^ to resect orthotopically implanted GBM tumors and create space to apply the therapeutic thermogels. Animals were anesthetized with ketamine 100 mg/kg/xylazine 10 mg/kg, by intraperitoneal (I.P.) injection, and a 7 mm incision was made in the midline along the previous surgical scar. A high-speed drill (Stoelting) was used to thin the skull area centered on the side of tumor implant to obtain a 3 mm diameter circular cranial window exposing the brain. A biopsy punch (7 mm long, 2 mm Ø, World Precision Instrument, WPI) was inserted 3 mm deep into the tissue to cut the brain tumor and then the explant was removed. The dried cavity void hosted 10 μL of THG or THG@SiO_2_-TMZ or THG@PCL-TMZ. In view of 3R-Reduction, only THG drug-free gel-based formulation was used to set the control group, as no difference in toxicity was detected in vitro among the drug free agents. The cranial and dural window were repaired using Duraform (Codman Neuro), a fibrin based dural substitute. The skin wound was then closed with surgical glue (3Mvetbond) animals allowed to recover. Paracetamol (150–300 mg/kg in water) was administered ad libitum following the operative procedures. Animals were monitored to awaken following surgery and did not display any signs of distress. Weight and behavior were monitored over time (Fig. S2).

### Bioluminescence imaging (BLI)

In vivo bioluminescence images of tumor-implanted mice were obtained using the IVIS Spectrum System (Perkin Elmer). Before imaging, mice were anesthetized as described previously, and D-luciferin (XenoLight D-Luciferin-K + Salt Bioluminescent Substrate 15 mg/mL, Perkin Elmer) was administered by I.P. injection, at a dose of 30 mg/kg and allowed to distribute for 10 min under isoflurane 2% + 2 Lt/min oxygen anesthesia. Animal were positioned in prone position into the IVIS chamber and kept at 37 °C during 1-min acquisition. Bioluminescence was captured as the absolute total flux (photons/sr/cm2−luminescence = Lum). Luminescence was normalized to baseline measurements and recorded at day 7, 14, 21, where baseline values were detected 2 days after resection and treatment initiation, to allow mice to recover from previous surgical procedures. Region of interest analysis was performed using Living Image Software to determine the light emitted (relative counts) from the tumor brain. The mean ± SD of the weekly (x) difference over base line signals (Lum(tx)−Lum (t0)/Lum (t0) of light emission (Lum) was plotted for each mouse.

### Statistical analysis

Sample size for the in vivo study was calculated to achieve significance (p < 0.05) with a power of 80% alpha 0.05. Statistical analysis was performed using GraphPad Prism 8.0 software (GraphPad Software, USA); two-ways ANOVA or Kruskal–Wallis test for multiple group variance comparison and Student’s t-test were used. All data shown are mean ± SEM. The difference was considered statistically significant if p < 0.05.

## Supplementary Information

Supplementary Figures.

## Data Availability

All data generated or analyzed during this study are included in this published article (and its supplementary information files). The datasets generated and/or analyzed during the current study are not publicly available but are available from the corresponding author on reasonable request.

## Abbreviations

ARRIVE: Animal Research: Reporting of In Vivo Experiments
BBB: Blood Brain Barrier
βGP: β-Glycerophosphate
BLI: Bioluminescence Imaging
CED: Convention Enhanced Delivery
CNT: Carbon nanotubes
DCM: Dichloromethane
DI: Deionized water
FDA: Food and Drug Administration
GBM: Glioblastoma Multiforme
H&E: Hematoxylin/Eosin stain
HPLC: High Performance Liquid Chromatography
IC50: Inhibitory dose 50%
I.P.: Intraperitoneal
IVIS: In Vivo Imaging System
Lum: Luminescence
MeOH: Methanol
MSNP: Mesoporous nanoparticles
MWCO: Molecular weight cutoff
PBS: Phosphate Buffer Saline
PEG: Polyethylene glycol
PLC: Polycaprolactone
SEM: Standard Error of Mean
THG: Chitosan-β-glycerophosphate-based thermogel
THG@PCL: Chitosan-β-glycerophosphate-based gel containing PLC microparticles
THG@PCL-TMZ: TMZ-loaded Chitosan/βGP thermogels with PLC microparticles
THG@SiO_2_: Chitosan/βGP-based gel containing mesoporous SiO_2_ nanoparticles
THG@SiO_2_-TMZ: TMZ-loaded Chitosan/βGP thermogels containing mesoporous SiO_2_ nanoparticles
TLS: Toscana Life Sciences
TMZ: Temozolomide
TUNEL: Terminal dUTP Nick End Labeling
VMD: Volume Mean Diameter (VMD)
WPI: World Precision Instrument
