# Supplementary material for: Loco-regional treatment with temozolomide-loaded thermogels prevents glioblastoma recurrences in orthotopic human xenograft models
Source: Sci Rep. 2023 Mar 21;13:4630. doi: 10.1038/s41598-023-31811-5 (PMC10030813; doi:10.1038/s41598-023-31811-5)
Supplement: Supplementary file 1 — Supplementary Figures. [file 41598_2023_31811_MOESM1_ESM.pdf]

Supplementary file

Loco-regional treatment with temozolomide-loaded thermogels prevents glioblastoma recurrences in orthotopic human xenograft models.

Lisa Gherardini[1,#], Veronica Vetri Buratti[2,#], Mirko Maturi[2], Giovanni Inzalaco[1,3,4], Erica Locatelli[2], Letizia Sambri[2], Sara Gargiulo[1], Virginia Barone[5], Denise Bonente[5,6], Eugenio Bertelli[5], Silvia Tortorella[2], Lorenzo Franci[1,3], Antonio Fioravanti[7], Mauro Comes Franchini[2,*], Mario Chiariello[1,3,*]

[1]Istituto di Fisiologia Clinica (IFC), Consiglio Nazionale delle Ricerche (CNR), Via Fiorentina 1, 53100, Siena, Italy; email: lisa.gherardini@cnr.it; sara.gargiulo@cnr.it

[2]Department of Industrial Chemistry "Toso Montanari" University of Bologna Viale Risorgimento 4, 40126, Bologna, Italy; email: veronica.vetri2@unibo.it; mirko.maturi2@unibo.it; erica.locatelli2@unibo.it; letizia.sambri@unibo.it; silvia.tortorella2@unibo.it

[3]Core Research Laboratory (CRL), Istituto per lo Studio, la Prevenzione e la Rete Oncologica (ISPRO), Via Fiorentina 1, 53100, Siena, Italy

[4]University of Siena, Via Banchi di Sotto 55, 53100, Siena Italy; email: inzalaco@student.unisi.it

[5]Department of Molecular and Developmental Medicine, University of Siena, Via Aldo Moro 2, 53100, Siena, Italy; email: virginia.barone@unisi.it; mail eugenio.bertelli@unisi.it

[6]Department of Life Sciences, University of Siena, 53100, Siena, Italy; email: denise.bonente@student.unisi.it

[7]Ospedale Maggiore (ASST) Largo Ugo Dossena 2, 26013, Crema, Italy; email: antonio.fioravanti@asst-cremona.it

[#]These authors contributed equally to the work.

*Correspondence:

Prof. Mauro Comes Franchini, PhD, Department of Industrial Chemistry "Toso Montanari", University of Bologna, Viale Risorgimento 4, 40126, Bologna, Italy Ph: +39 051 2093631 email: mauro.comesfranchini@unibo.it;

Mario Chiariello, MD, PhD, Istituto di Fisiologia Clinica (IFC), Consiglio Nazionale delle Ricerche (CNR), Via Fiorentina 1, 53100, Siena, Italy; Ph: +39057232174, email: mario.chiariello@cnr.it

Supplementary Figures

Fig S1

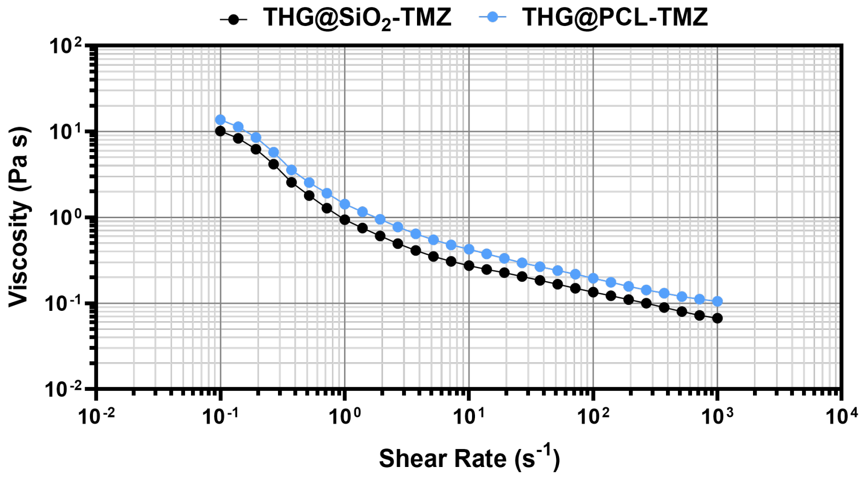

Fig. S1: Rotational viscosity of THG@SiO2-TMZ and THG@PCL-TMZ as a function of the applied shear rate.

Fig S2

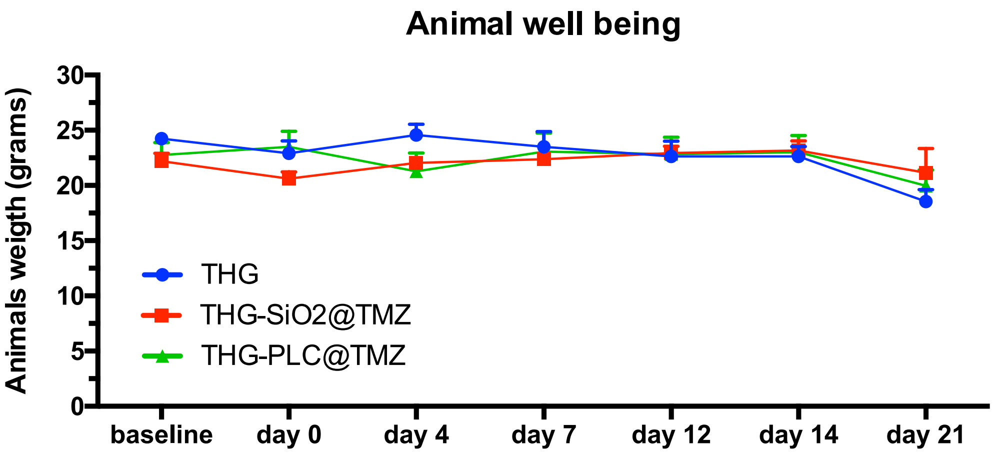

Fig. S2: The general well-being of the animals in the study was monitored by measuring the body weight from the start of the procedures up to the sacrifice. No statistical difference was detected among groups (ANOVA p value= 0,5527).

Fig S3

	[TMZ] (µg/mL)	Peak Area (a.u.)
Std 1	11,77	838,89
Std 2	23,54	1679,0
Std 3	47,07	3416,3
Std 4	94,14	6852,0
Std 5	176,5	12555

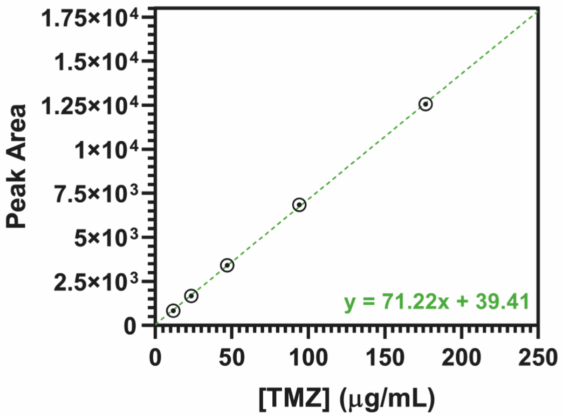

Fig. S3: Quantification of TMZ via HPLC. Left: TMZ concentration and the corresponding HPLC peak area for the calibration samples. Right: calibration curve with the corresponding curve equation.
